# Amorphous flexible covalent organic networks containing redox-active moieties: a noncrystalline approach to the assembly of functional molecules†
†Electronic supplementary information (ESI) available: Experimental methods, summary of previous studies, TG curves, ^13^C NMR spectra, molecular structures, UV-Vis-NIR spectra, SEM, AFM, and TEM images, CV curves, and reproducibility of the electrochemical properties. See DOI: 10.1039/d0sc01757d


**DOI:** 10.1039/d0sc01757d

**Published:** 2020-06-10

**Authors:** Jumpei Suzuki, Akira Ishizone, Kosuke Sato, Hiroaki Imai, Yu-Jen Tseng, Chi-How Peng, Yuya Oaki

**Affiliations:** a Department of Applied Chemistry , Faculty of Science and Technology , Keio University , 3-14-1 Hiyoshi, Kohoku-ku , Yokohama 223-8522 , Japan . Email: oakiyuya@applc.keio.ac.jp; b Department of Chemistry , National Tsing Hua University , Hsinchu 30013 , Taiwan

## Abstract

The organization states of functional molecules have a significant impact on the properties of materials. A variety of approaches have been studied to obtain well-organized molecular assemblies. The present work shows a new non-organized state of isolated and dispersed functional molecules in amorphous flexible covalent organic networks. Redox-active quinone molecules are embedded in the amorphous network polymers. Consecutive reactions between benzoquinone (BQ) and linker molecules generate random network structures through polymerization at different rates and in multiple directions. The low-crystalline stackings of the amorphous network polymers facilitate the formation of nanoflakes through exfoliation in dispersion media. Enhanced electrochemical performances, one of the highest specific capacities in recent studies, were achieved by efficient redox reactions of the quinone moiety. The present noncrystalline approach, low-crystalline stacking of designer amorphous covalent organic networks, can be applied to construct similar nanostructured polymer materials containing functional units.

## Abstract 2

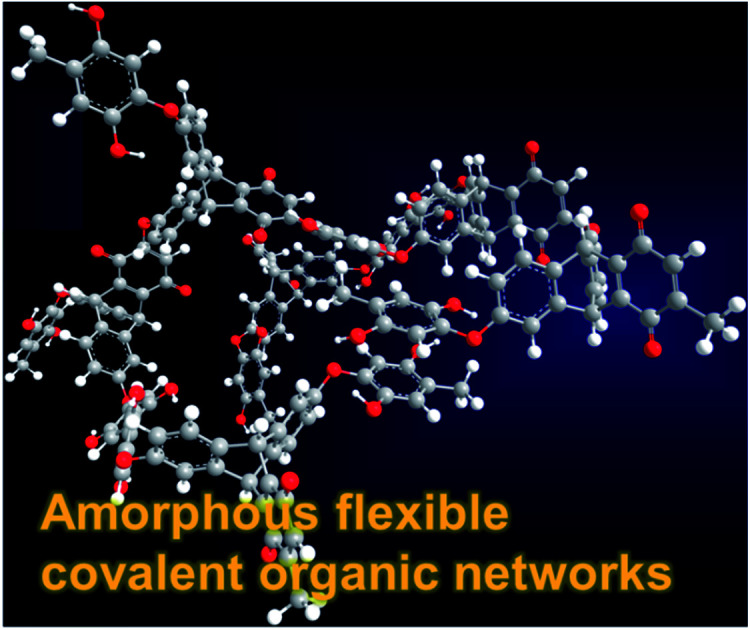
Amorphous flexible covalent organic networks containing functional molecules were synthesized by consecutive and multiple reactions at different rates and in multiple directions.

## Introduction

**Fig. 1:**
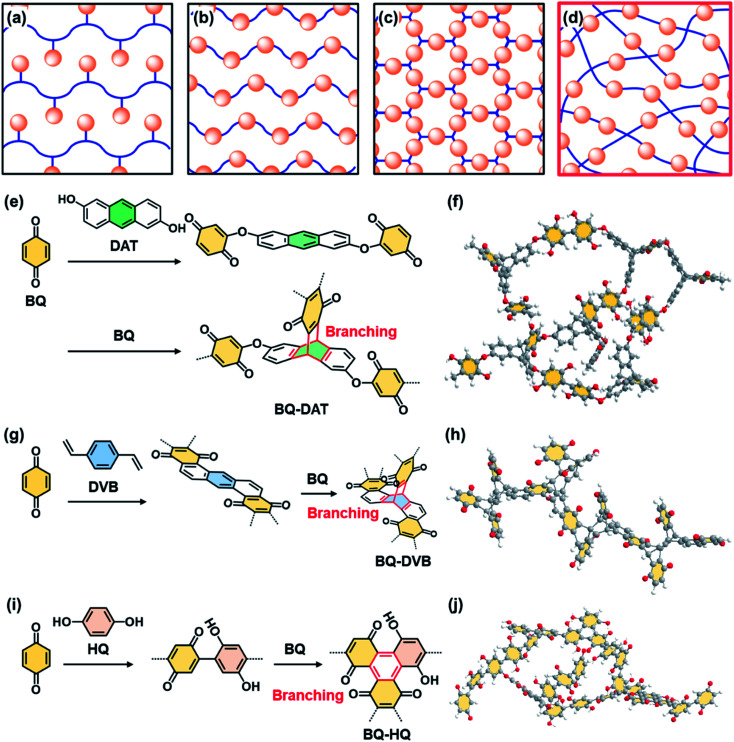
Schematic illustrations and molecular design of amorphous network polymers containing redox-active BQ moieties. (a–d) Assembly states of the functional molecules (orange spheres) in side-chain polymers (a), main-chain polymers (b), framework structures (c), and present amorphous flexible covalent organic networks (d). (e–j) Molecular design (e, g and i) and schematic illustration (f, h and j) of BQ–DAT (e and f), BQ–DVB (g and h), and BQ–HQ (i and j) amorphous covalent organic networks.

Functional molecules are organized in side- and main-chain polymers,^1^,^2^ self-assembled nanostructures,^3^,^4^ layered materials,^5^,^6^ and crystalline frameworks^7^–^11^ for the development of organic materials (Fig. 1a–c). The organized and orientated states have a significant impact on the dynamic properties.^1^–^6^,^11^ Here we show the potential of a new non-ordered assembly state, amorphous covalent organic network containing isolated and dispersed functional molecules (Fig. 1d). Functional molecules in the isolated and dispersed states can possess different properties compared with those in bulky aggregated, highly crystalline, and rigid states. For example, the isolated and dispersed redox-active molecules embedded in the flexible covalent organic network have potential for efficient electrochemical redox reactions leading to high specific capacity because the charge carrier and electrolyte easily penetrate the functional units in the flexible network. Moreover, the low-crystalline stacking of the network polymer facilitates exfoliation into nanostructures exhibiting redox-active moieties on the surface. Here we designed and synthesized three amorphous covalent organic networks containing a redox-active benzoquinone (BQ) moiety (Fig. 1e–j). The aggregates were easily exfoliated into nanostructures in organic media. The nanoflakes of the amorphous flexible covalent organic networks showed one of the highest specific capacities in recent reports about quinone-based supercapacitors in aqueous electrolytes (Fig. S1 and Table S1 in the ESI†).^12^–^23^

The covalent organic networks are designed and synthesized by simultaneous multiple reactions at different rates and in multiple growth directions (Fig. 1e–j). In general, polymer synthesis is intended to control the shape, molecular weight, and sequence.^24^–^27^ On the other hand, an amorphous network with a random sequence and direction is not a main target of polymer chemistry. The simultaneous and multiple reactions facilitate the generation of amorphous networks containing functional units (Fig. 1d, e, g and i). If such amorphous network polymers form aggregates, the low-crystalline stacking different from the rigid crystalline states can facilitate exfoliation into nanostructured materials. In recent years, crystalline polymeric frameworks, such as covalent-organic frameworks (COFs), have attracted much interest.^7^–^11^ Our previous work suggested that low-crystalline stacking states with more flexibility have an advantage for smooth exfoliation into nanostructures, such as two-dimensional (2D) amorphous network polymers.^28^ In the present work, the pristine low-crystalline stackings of new three-dimensional (3D) amorphous network polymers were exfoliated into nanostructures for their applications.

Quinone derivatives have been studied for application in organic energy storage, such as lithium-ion batteries and supercapacitors.^12^–^23^,^29^–^39^ Two-electron redox reactions of quinone derivatives enable high theoretical specific capacity, such as 496 mA h g^–1^ for BQ and 339 mA h g^–1^ for naphthoquinone (NQ). However, bulk crystals of these low-molecular-weight compounds are not used as an active material for electrodes because of the low conductivity and dissolution in electrolyte solution. In addition, the molecules inside of bulk and condensed states are not efficiently used for redox reactions because of the longer diffusion distance of the charge carriers and electrolytes. Stable redox reactions with a high specific capacity were reported on main- and side-chain polymers containing quinone moieties.^34^–^39^ Another approach is adsorption of quinone derivatives on porous conductive solids, such as nanocarbons.^12^–^21^ Our group prepared composites of quinone derivatives and conductive polymers with controlled morphologies from nanoscopic to macroscopic scales.^22^,^23^,^33^ However, the highest specific capacity was 136 mA h g^–1^ at 1 A g^–1^ in recent reports about supercapacitors in an aqueous electrolyte, to the best of our knowledge (Fig. S1 in the ESI†).^12^ New design strategies of molecules and nanostructures are required to promote redox reactions more efficiently for enhanced electrochemical performances. In the present work, the isolated and dispersed BQ moiety in the nanostructures of the amorphous covalent organic networks showed one of the highest specific capacities, such as 185 mA h g^–1^ at 0.2 A g^–1^ in recent studies (Fig. S1 in the ESI†). The new design strategy of polymers and nanostructures can be applied to other functional molecules for the improvement of their properties.

## Results and discussion

### Molecular design and synthesis

The amorphous covalent organic networks containing the BQ moiety were designed and synthesized with three types of crosslinkers, such as 1,9-dihydroxyanthracene (DAT), 1,4-divinylbenzene (DVB), and hydroquinone (HQ) (Fig. 1e–j). BQ as a target monomer shows different types of reactions with these cross-linkers. BQ and DAT initially form an ether bond at the *α* position of BQ with electrophilic nature (Fig. 1e). The Diels–Alder reaction between the anthracene ring as 4π and BQ as 2π induces branching through the formation of the triptycene moiety. A previous study showed the Diels–Alder reaction of anthracene and BQ derivatives.^40^ As these two reactions consequently proceed at different rates and in multiple directions, an amorphous network polymer of BQ and DAT is obtained (Fig. 1e and f). Two types of Diels–Alder reactions consecutively proceed between BQ and DVB (Fig. 1g). The first Diels–Alder reaction generates an anthracene moiety through the recovery of the conjugation *via* dehydrogenation by free BQ. The anthracene moiety serves as 4π for the subsequent Diels–Alder reaction with branching. In this manner, the consecutive Diels–Alder reactions with branching form an amorphous network polymer (Fig. 1g and h). BQ directly forms a C–C bond with HQ (Fig. 1i). The dimerized BQ–HQ unit as 4π and BQ as 2π provide the benzene ring as the branching unit *via* the Diels–Alder reaction. Therefore, the amorphous network polymer is obtained by these reactions (Fig. 1i and j). The Diels–Alder reaction was applied to polymerization of quinone derivatives.^41^ In addition, bottom-up syntheses of nanocarbons using Diels–Alder reactions were studied to obtain well-defined graphene analogues.^42^ However, the formation of amorphous network polymers and their applications were not studied in previous studies. The present molecular design implies that simultaneous multiple reactions at different rates and in multiple directions enable the formation of amorphous network polymers containing randomly distributed quinone moieties.

The three types of amorphous network polymers were synthesized by mixing of the two monomers. Powder of 2.5 mmol BQ and 0.5 mmol DAT was mixed and then heated at 120 °C for 48 h in an autoclave. A mixture of 40 mmol BQ powder and 8.0 mmol DVB liquid was reacted at 110 °C for 48 h in an autoclave. Powder of 27 mmol BQ and 3.0 mmol HQ was dissolved in 1 mol dm^–3^ sulfuric acid (H_2_SO_4_) and then maintained at 25 °C for 48 h under ambient pressure. The resultant precipitates were rinsed and then dried under vacuum at 200 °C for 16 h to remove the monomers and oligomers. The detailed procedure is described in the ESI.†

### Structures of amorphous covalent organic networks

**Fig. 2:**
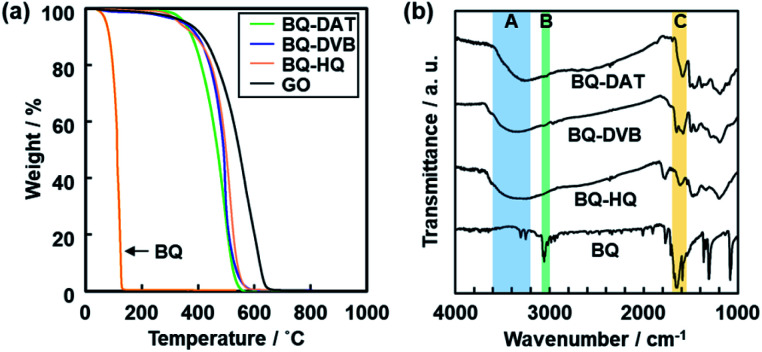
Molecular structures of amorphous covalent organic networks. (a) TG curves of the BQ–DAT, BQ–DVB, BQ–HQ, monomer BQ, and reference GO. (b) FR-IR spectra of the BQ–DAT, BQ–DVB, BQ–HQ, and monomer BQ. The absorption bands A–C correspond to the stretching vibrations of C=O, C–H, and O–H bonds. The more detailed peak assignments are described in Fig. S2 in the ESI.†

A black powder was obtained after the polymerization. The polymers of BQ and DAT (BQ–DAT), BQ and DVB (BQ–DVB), and BQ and HQ (BQ–HQ) showed weight loss around 500 °C under air atmosphere on the thermogravimetric (TG) curves (Fig. 2a). A similar TG curve was observed for commercial graphene oxide (GO) and an amorphous network polymer of BQ and pyrrole (Py) in our previous work (Fig. 2a).^28^ In contrast, the weight loss was observed below 300 °C for the monomers except DAT (Fig. S2 in the ESI†). The other reference compounds, such as a charge-transfer (CT) complex of BQ and HQ (CT–BQ/HQ) and polymer of DVB, showed different weight loss behavior (Fig. S2 in the ESI†). The results imply that BQ–DAT, BQ–DVB, and BQ–HQ form polymerized network structures.

**Fig. 3:**
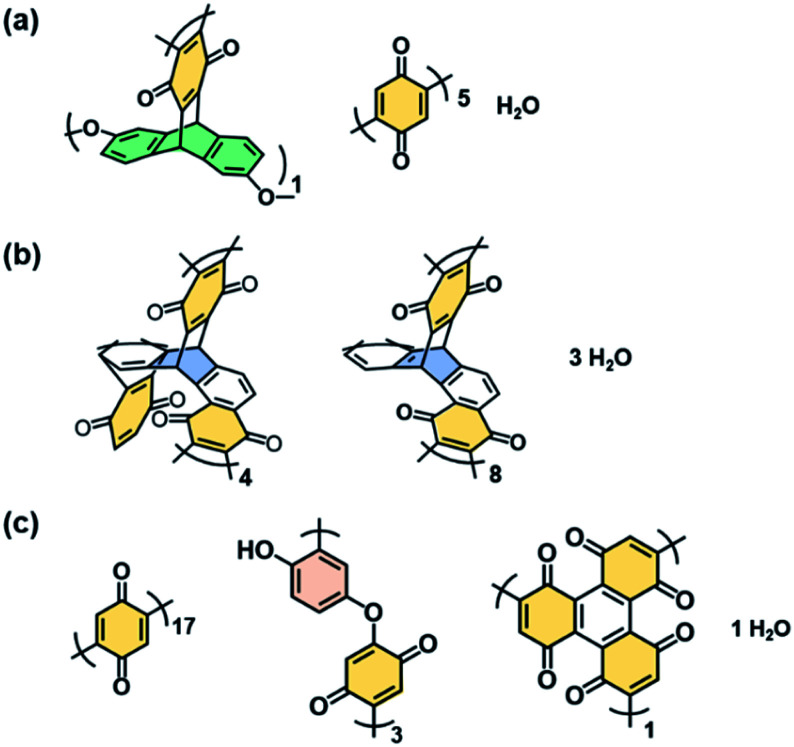
Estimated structure units and compositions of BQ–DAT (a), BQ–DVB (b), and BQ–HQ (c) polymers estimated from CHN elemental analysis in Table S2.†

Broadened absorption bands corresponding to C=O and O–H stretching vibration were observed on the Fourier-transform infrared (FT-IR) spectra (the bands A and C in Fig. 2b), whereas absorption of the C–H stretching vibration was not clearly observed after the polymerization (the band B in Fig. 2b). The results indicate that the BQ moieties are embedded in the polymer network structures. The other detailed peak assignments supported the formation of the designed structures as shown in Fig. 1 (Fig. S2 in the ESI†). ^13^C solid-state nuclear magnetic resonance (NMR) spectroscopy also supported the formation of the designed network polymers (Fig. S3 in the ESI†). The weight ratio of C, H, and O was estimated to be C : H : O = 69.1 : 3.60 : 27.3 for BQ–DAT, 75.2 : 3.63 : 21.1 for BQ–DVB, and 67.6 : 3.13 : 28.8 for BQ–HQ by CHN elemental analysis. Based on the composition, the network polymers contained the reduced state of BQ, namely HQ, 41.7% for BQ–DAT, 68.8% for BQ–DVB, and 40.0% for BQ–HQ. These compositions are consistent with the designed structures in Fig. 1 (Fig. 3 and Table S2 in the ESI†).

The rigid and soft natures are changed by the degree of cross-linking and linking state of the functional units (Fig. 1e–j and 3). Since the BQ units are embedded in the BQ–DVB network like a ladder polymer (Fig. 3b), BQ–DVB is regarded as a more rigid polymer network. In contrast, more flexible networks are formed for BQ–DAT and BQ–HQ because of the linear BQ chain between the cross-linking points (Fig. 3a and c).

### Low-crystalline layered structures and their exfoliation into nanostructures

**Fig. 4:**
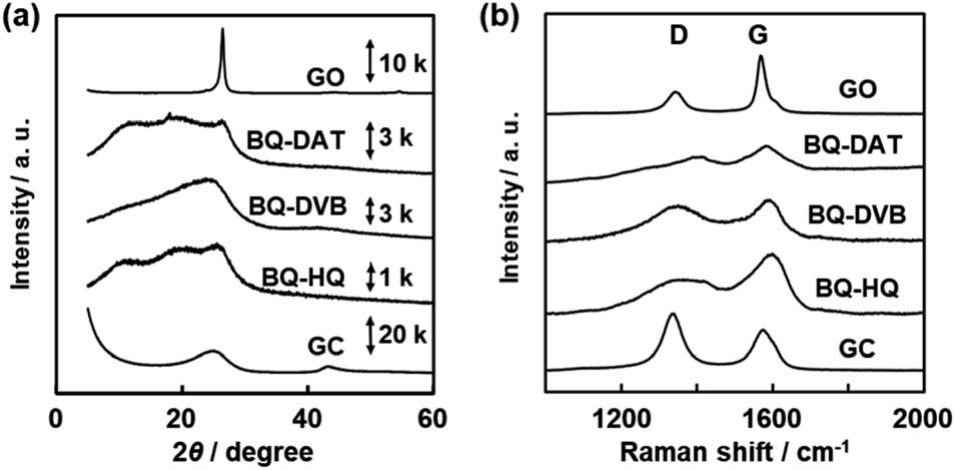
XRD patterns (a) and Raman spectra (b) of the BQ–DAT, BQ–DVB, BQ–HQ, monomer BQ, and commercial GO and GC.

The amorphous network polymers formed aggregates with low crystallinity (Fig. 4). The BQ–DAT, BQ–DVB, and BQ–HQ polymers showed a broadened weak peak around 2*θ* = 25° on the X-ray diffraction (XRD) pattern (Fig. 4a). A similar broadened peak was observed for commercial glassy carbon (GC) and a BQ–Py network polymer (Fig. 4a),^28^ whereas GO had a sharpened peak. The lattice spacing around *d* = 0.356 nm (2*θ* = 25°) corresponds to the weak periodicities of the stacked graphitic domains. The peak weakening and broadening of the BQ–DAT, BQ–DVB, and BQ–HQ polymers indicate the low-crystalline states of the network structures and their stackings. Raman spectra showed broadened D and G bands characteristic of sp^3^ and sp^2^ carbons around 1350 cm^–1^ and 1580 cm^–1^, respectively (Fig. 4b).^43^ The peak intensity (*I*) ratio of G to D bands (*I*_G_/*I*_D_) was 1.21 for BQ–DAT, 1.10 for BQ–DVB, and 1.74 for BQ–HQ. These *I*_G_/*I*_D_ values were smaller than that of commercial GO (2.52) and larger than that of commercial GC (0.73). The Raman analysis implies the formation of a graphitic network with the branching units *via* sp^3^ bonds (Fig. 1). The peak broadening is ascribed to the low-crystalline network structure. These polymers showed a broadened absorption edge from the UV-Vis to the near infrared (NIR) region (Fig. S4 in the ESI†). The entire absorption from the visible to the NIR region is ascribed to the extension of the conjugation length. Since the BQ moiety partially reduced to HQ in these polymers (Fig. 3 and Table S2 in the ESI†), the appearance of absorption in the NIR region is ascribed to the homogenously distributed inter- and intra-molecular CT complexes between the BQ and HQ moieties in the network. In this manner, the amorphous covalent organic networks of BQ–DAT, BQ–DVB, and BQ–HQ formed low-crystalline stackings.

**Fig. 5:**
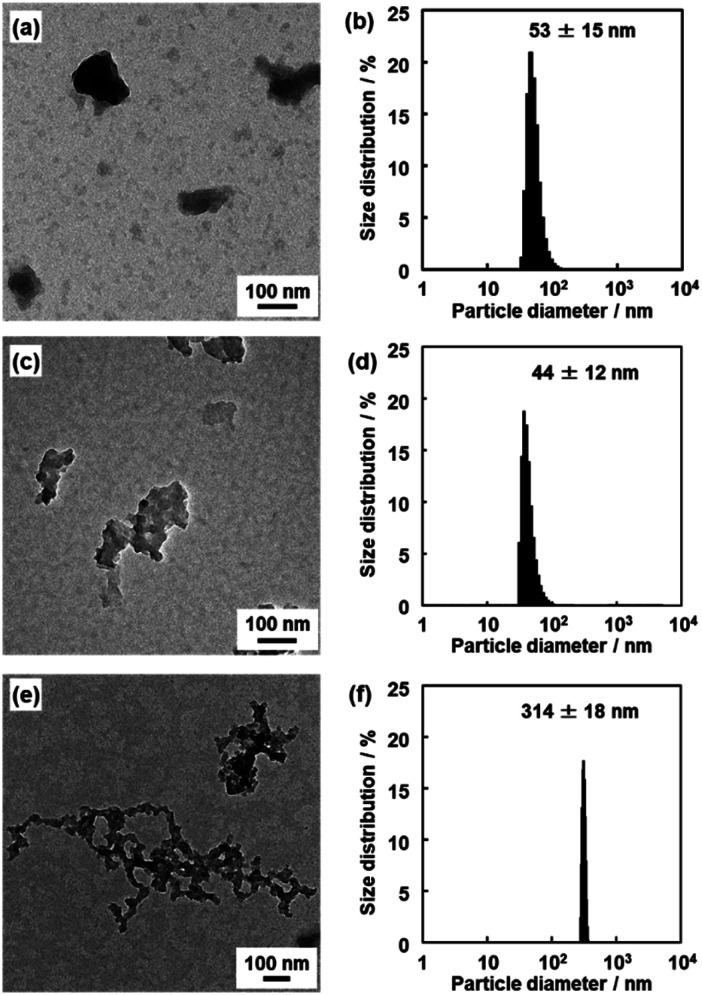
TEM images (a–c) and DLS charts (d–f) of the BQ–DAT (a and d), BQ–DVB (b and e), and BQ–HQ (c and f) polymers after the exfoliation into nanostructures.

The nanoflakes of these amorphous network polymers around 100 nm in size were obtained after dispersion in organic media (Fig. 5). The original BQ–DAT, BQ–DVB, and BQ–HQ polymers showed irregularly shaped particles around several micrometers in size (Fig. S5 in the ESI†). The bulk particles were exfoliated into nanoflakes after dispersion in certain organic media at 60 °C for 2 days, such as benzyl alcohol for BQ–DVB and BQ–DAT and ethanol for BQ–HQ. The average size of the polymer nanoflakes was 57 ± 30 nm in width and 19 ± 4.2 nm in thickness for BQ–DAT, 447 ± 16 nm in width and 8.1 ± 3.3 nm in thickness for BQ–DVB, and 263 ± 125 nm in width and 36 ± 16 nm in thickness for BQ–HQ estimated from the images of transmission electron microscopy (TEM) and atomic force microscopy (AFM) (Fig. 5a–c and S6 in the ESI†). The particle-size distribution was observed in a similar range by dynamic light scattering (DLS) measurement (Fig. 5d–f). Our previous studies indicated that DLS peak appeared with formation of nanostructures after the exfoliation in dispersion media.^28^,^44^ The lattice fringes were not observed on the high-resolution TEM (HRTEM) images (Fig. S7 in the ESI†). These results indicate that the nanoflakes of the amorphous network polymers were generated from their low-crystalline stackings through exfoliation. Although the branches are irregularly introduced into the network structure, the polymerization reactions including branching simultaneously proceed in the reaction system. The primary particles with a similar size form aggregates (Fig. S5 in the ESI†). The exfoliation treatment enables the dispersion of the homogeneous nanoflakes as the primary particles in organic media (Fig. 5).

### Electrochemical properties as a supercapacitor in an aqueous electrolyte

**Fig. 6:**
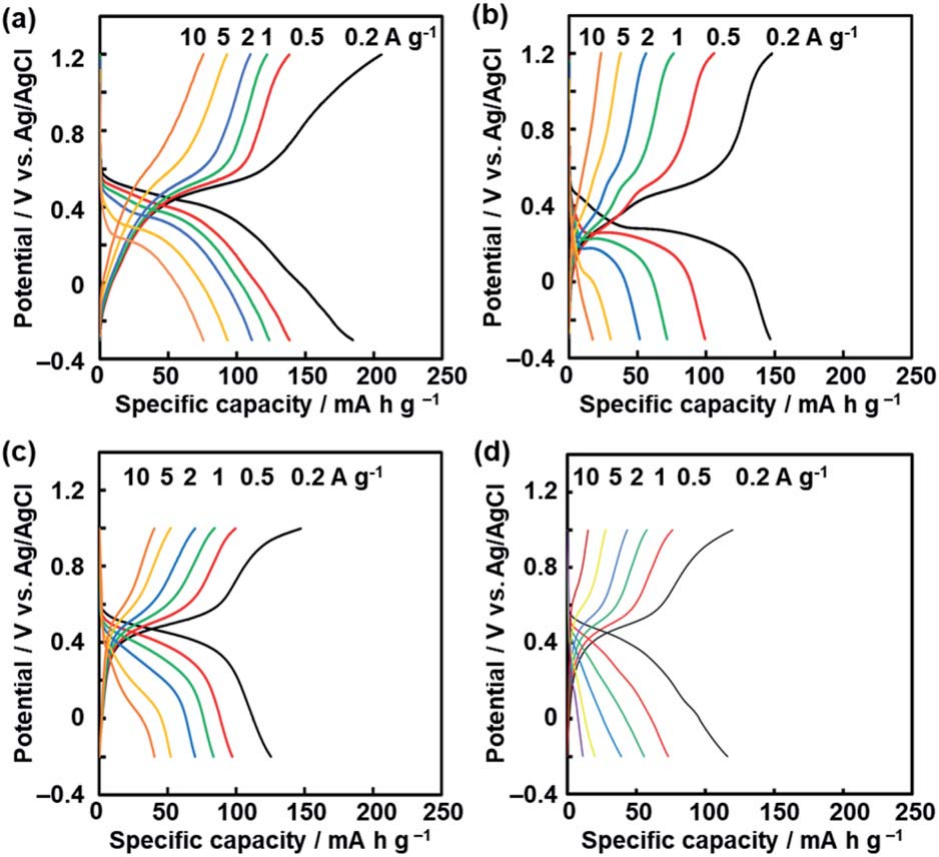
Charge–discharge curves of the BQ–DAT nanostructures (a), BQ–DVB nanostructures (b), and BQ–HQ (c) and more branched BQ–HQ polymers (d) before the exfoliation.

High specific capacity was observed for the BQ–DAT, BQ–DVB, and BQ–HQ polymers and nanostructured samples originating from the redox reactions of the quinone moiety in an aqueous electrolyte (Fig. 6). The polymer sample, acetylene black conductive carbon, and poly(vinylidene fluoride) (PVDF) were mixed at a ratio of 6 : 3 : 1 in weight. The electrochemical properties were measured in a beaker cell containing 1 mol dm^–3^ sulfuric acid (H_2_SO_4_) using platinum as the counter electrode and Ag/AgCl as the reference electrode. The charge–discharge measurement showed a plateau originating from the redox reactions of the quinone moieties around 0.4 V *vs.* Ag/AgCl (Fig. 6). The broadened peaks of the cathodic and anodic currents were observed in the range 0.1–0.6 V by cyclic voltammetry (CV) (Fig. S8 in the ESI†). BQ and NQ moieties have redox peaks around 0.5 V and 0.3 V, respectively. Therefore, the redox reactions of the BQ and NQ moieties proceed in the network polymers (Fig. 1).

The BQ–DAT and BQ–DVB nanostructures showed a specific capacity of 185 mA h g^–1^ and 147 mA h g^–1^ at 0.2 A g^–1^, respectively (Fig. 6a and b). These specific capacities were reproducible for five different samples (Fig. S9 in the ESI†). In particular, the specific capacity of the BQ–DAT nanostructures is one of the highest performances compared with recent reports about quinone-based supercapacitors in aqueous electrolytes (Fig. S1 and Table S1 in the ESI†).^12^–^23^ In contrast, the bulk aggregates before the exfoliation showed a larger overpotential and lower specific capacity (Fig. S8 in the ESI†). After the exfoliation, the quinone moieties exposed on the surface of the BQ–DAT and BQ–DVB nanostructures are efficiently utilized for the redox reactions. A recent study suggested that the CT complex promotes charge–discharge reactions with improved conductivity.^45^ In the present work, the inter- and intra-molecular CT complexes are homogeneously distributed in the polymer networks at the molecular level. In addition, a higher specific capacity is achieved by the more flexible network of the BQ–DAT polymer originating from BQ moieties linked with ether bonds. Since the more flexible structure enables the penetration of the electrolytes in the network, the higher utilization rate of the BQ unit contributes to improving the specific capacity of BQ–DAT. In a previous study, the redox moiety in a more flexible state showed a higher specific capacity.^46^ The characteristic structures play important roles for enhanced electrochemical performances.

The specific capacity of the BQ–HQ polymer before the exfoliation treatment was 125 mA h g^–1^ at 0.2 A g^–1^ (Fig. 6c). However, side reactions were observed on the BQ–HQ nanoflakes at potential around –0.2 V (Fig. S8 in the ESI†), because of the hexahydroxytriphenylene moiety in the network structure. The degree of cross-linking was controlled by changing the synthetic conditions. A more branched network of the BQ–HQ polymer was obtained by solvent-free direct mixing of BQ and HQ and heating at 190 °C for 48 h (Fig. S10 in the ESI†). The more branched BQ–HQ network showed a lower specific capacity compared with the less branched one (Fig. 6d and S10 in the ESI†). The results support that the flexible structure originating from the less branched network has potential for enhanced electrochemical performances. Moreover, the dynamic properties of the functional units can be tuned by the flexibility originating from the cross-linkage of amorphous covalent organic networks.

## Conclusions

Amorphous flexible covalent organic networks containing functional unit molecules were synthesized by consecutive and multiple reactions at different rates and in multiple directions. Nanostructures were obtained by exfoliation of the bulky aggregates after dispersion in organic media because of the low-crystalline stacking of the amorphous network polymers. The flexibility originating from the branching in the network polymer plays important roles for the properties. The nanostructure of flexible BQ–DAT showed one of the best performances in recent studies about quinone-based supercapacitors in aqueous electrolytes. The quinone moieties exposed on the surface of the nanostructures are effectively used for redox reactions. The present noncrystalline approach, low-crystalline stacking of amorphous network polymers, can be applied to materials design based on a variety of functional molecules.

## Conflicts of interest

There are no conflicts to declare.

## Supplementary Material

Supplementary information
