# Supplementary material for: Amorphous flexible covalent organic networks containing redox-active moieties: a noncrystalline approach to the assembly of functional molecules
Source: Chem Sci. 2020 Jun 10;11(27):7003–8. doi: 10.1039/d0sc01757d (PMC7504977; doi:10.1039/d0sc01757d)
Supplement: Supplementary file 1 [file SC-011-D0SC01757D-s001.pdf]

Electronic Supplementary Material (ESI) for Chemical Science.
This journal is © The Royal Society of Chemistry 2020

Electronic Supplementary Information (ESI)

Amorphous flexible covalent organic networks containing redox-active moieties: A noncrystalline approach to assembly of functional molecules

Jumpei Suzuki,[a] Akira Ishizone,[a] Kosuke Sato,[a] Hiroaki Imai,[a] Yu-Jen Tseng,[b] Chi-How Peng,[b] Yuya Oaki*,[a]

[a] Department of Applied Chemistry, Faculty of Science and Technology, Keio University, 3-14-1 Hiyoshi, Kohoku-ku, Yokohama 223-8522, Japan.

[b] Department of Chemistry, National Tsing Hua University, Hsinchu 30013, Taiwan.

E-mail: oakiyuya@applc.keio.ac.jp

Contents

Experimental methods	P. S2
Previous works on energy storage using quinone derivatives (Fig. S1 and Table S1)	P. S4
TG curves and FT-IR spectra (Fig. S2)	P. S6
^{13}C Solid-state NMR spectra (Fig. S3)	P. S8
Estimated compositions (Table S2)	P. S9
UV-Vis-NIR spectra (Fig. S4)	P. S10
SEM images (Fig. S5)	P. S11
AFM images, their height profiles, and thickness distribution (Fig. S6)	P. S12
SAED patterns and HRTEM images (Fig. S7)	P. S13
CV and charge-discharge curves (Fig. S8)	P. S14
Reproducibility of the electrochemical properties (Fig. S9)	P. S15
Effects of the branching on the electrochemical properties (Fig. S10)	P. S16

Experimental methods

Syntheses of BQ-DAT, BQ-DVB, and BQ-HQ polymers and their nanostructures: Mixture of 40 mmol *p*-benzoquinone (BQ, TCI, 98.0 %) and 0.5 mmol 1,9-dihydroxyanthracene (DAT, TCI, 95.0 %) was heated at 120 °C for 48 h in a polytetrafluoroethylene (PTFE) autoclave sealed with stainless jacket. The resultant precipitates were rinsed with acetone. Then, the powder was dried under vacuum at 150 °C for 16 h to remove the oligomers and remaining monomers. The powder was dispersed in benzyl alcohol (Kanto, 99.0 %) under ultrasonication for 1.5 h. The resultant nanostructure of BQ-DAT was collected using a PTFE membrane filter 0.2 μm in pore size. The collected powder was dried at 60 °C for 16 h.

Powder of 40 mmol BQ and liquid of 8 mmol divinylbenzene (DVB, TCI, 50.0 %) were mixed and heated at 110 °C for 48 h in the autoclave. The resultant precipitates were rinsed with acetone and *N*-methyl-2-pyrolidone (NMP). Then, the resultant BQ-DVB polymer was vacuum-dried at 200 °C for 16 h to remove the oligomers, remaining monomers, and NMP. The powder was dispersed in benzyl alcohol under ultrasonication for 1.5 h. Then, the dispersion liquid was stirred at 60 °C for 2 days to obtain the nanostructures. The resultant nanostructures were collected and then dried by the methods same as those of BQ-DAT.

Powder of 27 mmol BQ and 3.0 mmol hydroquinone (HQ, TCI, 99.0 %) was dissolved in 1 mol dm^{-3} sulfuric acid (H_2SO_4, Kanto, 96.0 %) and then maintained at 25 °C for 48 h under ambient pressure. The resultant precipitates were rinsed with purified water and then dried under vacuum at 200 °C for 16 h to remove the oligomers. The powder was dispersed in ethanol under ultrasonication for 1.5 h. Then, the dispersion liquid was stirred at 60 °C for 2 days to obtain the nanostructures.

Structure characterization: Thermogravimetric analysis (TG, SII TG-DTA 7200) was performed under air atmosphere. The molecular structures were analyzed by Fourier-transform infrared (FT-IR, Jasco, FT-IR 4200) absorption spectroscopy and ^{13}C solid-state nuclear-magnetic resonance (NMR) spectroscopy. The powdered samples were analyzed by potassium bromide (KBr) method for FT-IR and magic angle spinning method for NMR. The compositions of the polymers were calculated by the results of CHN elemental analysis. The crystallinity of the polymers was studied by X-ray diffraction with Cu-Kα radiation (XRD, Bruker, D8 Advance). The powdered sample was filled in a low-background silicon sample holder without diffraction peaks in the measuring range. Raman spectroscopy (Renishaw, inVia Raman Microscope) was performed on the powered samples. The dried powder was set on a

silicon substrate and excited by laser 532 nm. UV-Vis-NIR spectra were obtained by a spectrophotometer with an integrated sphere (UV-Vis-NIR, JASCO V-670). The morphologies of the resultant polymers were observed by a field-emission scanning electron microscopy (FE-SEM, JEOL, JSM 7600F) and transmission electron microscopy (TEM, FEI Tecnai G2 Spirit and F20). The thickness of the nanostructures was measured by atomic force microscopy (AFM, Shimadzu, SPM-9700HT). The dispersion liquid was dropped on a collodion membrane for TEM observation and a cleaned silicon substrate for AFM observation. The particle-size distribution of the nanostructures in the dispersion liquid was estimated using dynamic light scattering (DLS, Otsuka electronics, ELSZ-2000ZS).

Electrochemical properties: A three-electrode setup in a twin-beaker cell was used for electrochemical measurements. The powdered samples of BQ-DAT, BQ-DVB and BQ-HQ were used as the active materials. 60 wt% of active material, 30 wt% of acetylene black as a conductive carbon, and 10 wt% of poly(vinylidene fluoride) (PVDF) as a binder were mixed to prepare the working electrode. The mixture was dispersed with a small amount of NMP to obtain the slurry. The slurry was dropped on a titanium mesh as a current collector and then dried 60 °C for overnight. Platinum and Ag/AgCl electrodes were used as counter and reference electrodes, respectively. 1 mol dm^{-3} sulfuric acid aqueous solution was used as electrolyte solution. Cyclic voltammetry was performed by a potentiostat (Princeton Applied Research, VersaSTAT 3) at scan rate 1 mV s^{-1}. Chronopotentiometry was measured by a charge-discharge measurement system (Hokuto Denko, HJ1001SD8) at the different current density.

Previous works about energy storage using quinone derivatives

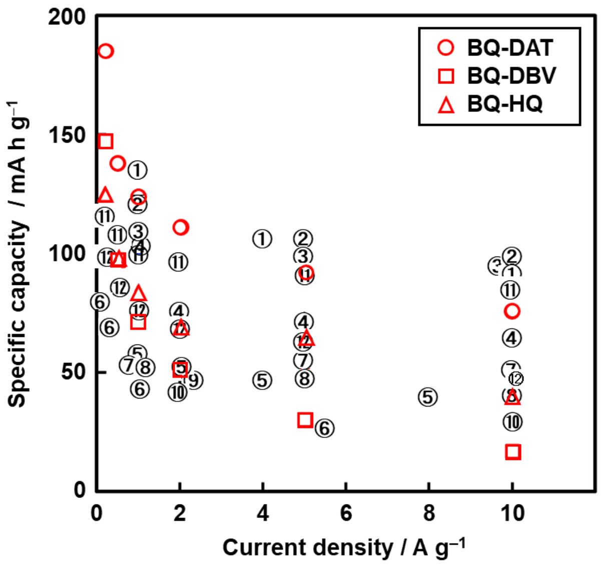

Fig. S1. Relationship between the current density and specific capacity reported in recent reports about quinone-based supercapacitor in aqueous electrolytes.[12–23] The numbers in the circular plots, materials, and references are summarized in Table S1 (P. S5).

These recent reports show the higher specific capacity compared with the other works. The BQ-DAT and BQ-DVB nanostructures in the present work showed the highest specific capacity at 0.2 and 0.5 A g^{-1} (the circles and square in Fig. S1).

Table S1 Previous reports about quinone-based supercapacitor in an aqueous electrolyte.

No.	Active material	Refs.
○	BQ-DAT polymer nanostructure (the present work)	–
□	BQ-DVB polymer nanostructure (the present work)	–
Δ	BQ-HQ polymer before the exfoliation (the present work)	–
1	Anthraquinone (AQ) on activated carbon (the present work)	12
2	HQ on graphene hydrogel	13
3	AQ on graphene	14
4	Bisphenol A adsorbed on graphene	15
5	Tert-butyl-hydroquinone adsorbed on graphene	16
6	AQ and tetrachlorohydroquinone on activated carbon	17
7	Lignin on reduced graphene oxide (r-GO)	18
8	2-aminoanthraquinone on graphene	19
9	Tetrachloro-1,4-benzoquinone on carbon	20
10	AQ grafted on activated carbon	21
11	2-chloro-5-methyl-1,4-benzoquinone incorporated in polypyrrole (PPy)	22
12	2,3-dichloro-1,4-naphthoquinone in PPy matrix	23

TG curves and FT-IR spectra

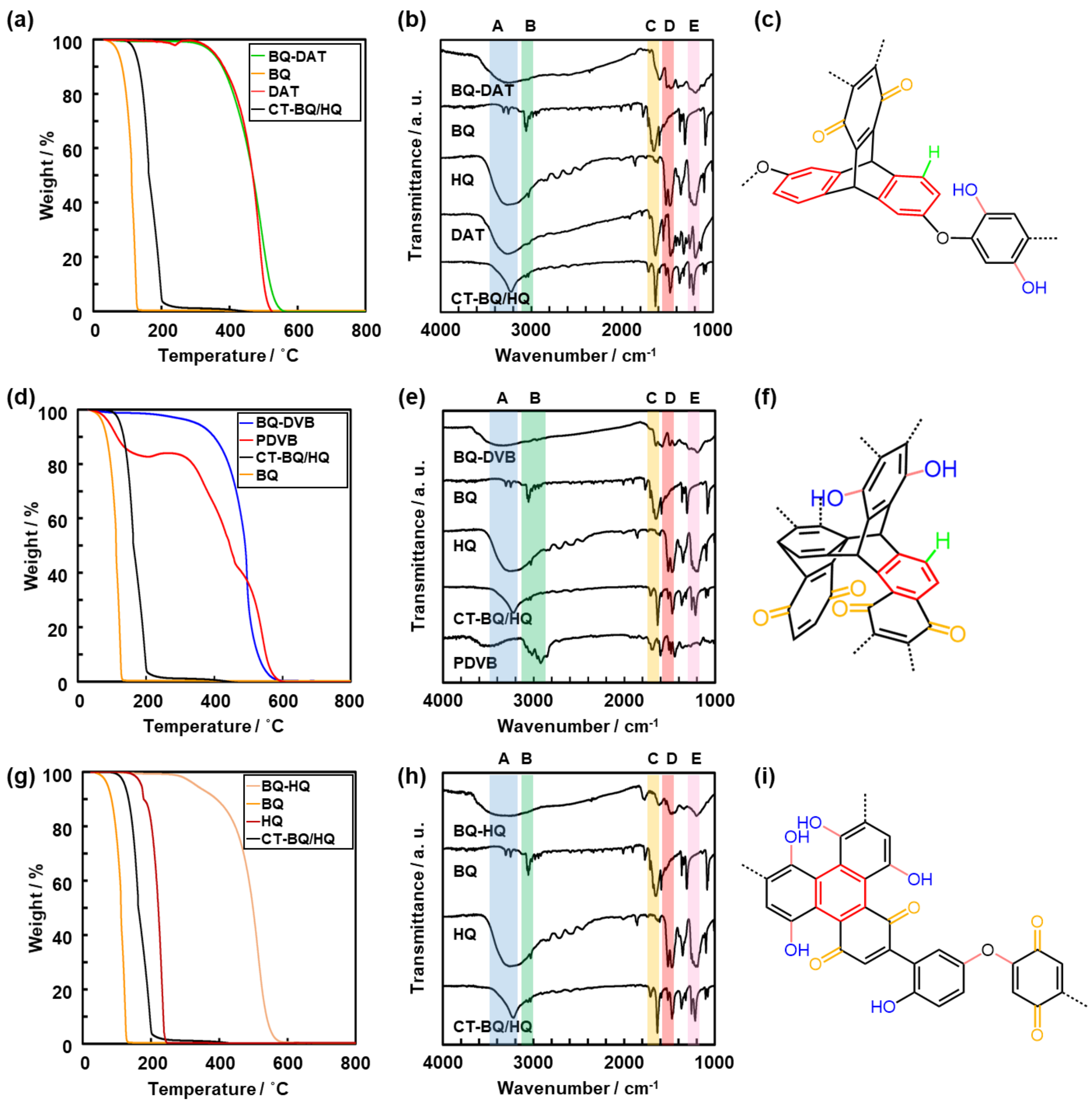

Fig. S2. TG curves (a,d,g), FT-IR spectra (b,e,h), and unit structures (c,f,i) of the BQ-DAT (a–c), BQ-DVB (d–f), and BQ-HQ (g–i) polymers and their reference samples, such as monomers.

The BQ-DAT showed the weight loss around 500 °C higher than that of BQ and the charge-transfer (CT) complex of BQ and HQ (CT-BQ/HQ) (Fig. S2a). Monomeric DAT also had the weight loss around 500 °C. The BQ-DVB showed the weight loss around 550 °C higher than that of BQ, HQ and CT-BQ/HQ (Fig. S2d). DVB was polymerized to obtain the reference sample. The polymerized DVB showed the different TG curve compared with BQ-DVB. The BQ-HQ showed the weight loss around 550 °C higher than that of BQ, HQ and CT-BQ/HQ (Fig. S2g).

The following absorption bands were observed for BQ-DAT, BQ-DVB, and BQ-HQ on the

FT-IR spectrum (Fig. S2b,e,h): the stretching vibration of O-H group (blue, A), the stretching vibration of C-H group (green, B), the stretching vibration of C=O group (yellow, C), the stretching vibration of C-C bond in aromatic ring (red, D), and stretching vibration of C-O bond in the phenolic hydroxy group (pink, E). Appearance of these peaks is consistent with the designed and estimated structures, as shown in Fig. S2c,f,i. The 13C NMR spectra also supported formation of these structures (Fig. S3). In addition, the peak broadening implies formation of the polymerized structures.

^{13}C solid-state NMR spectra

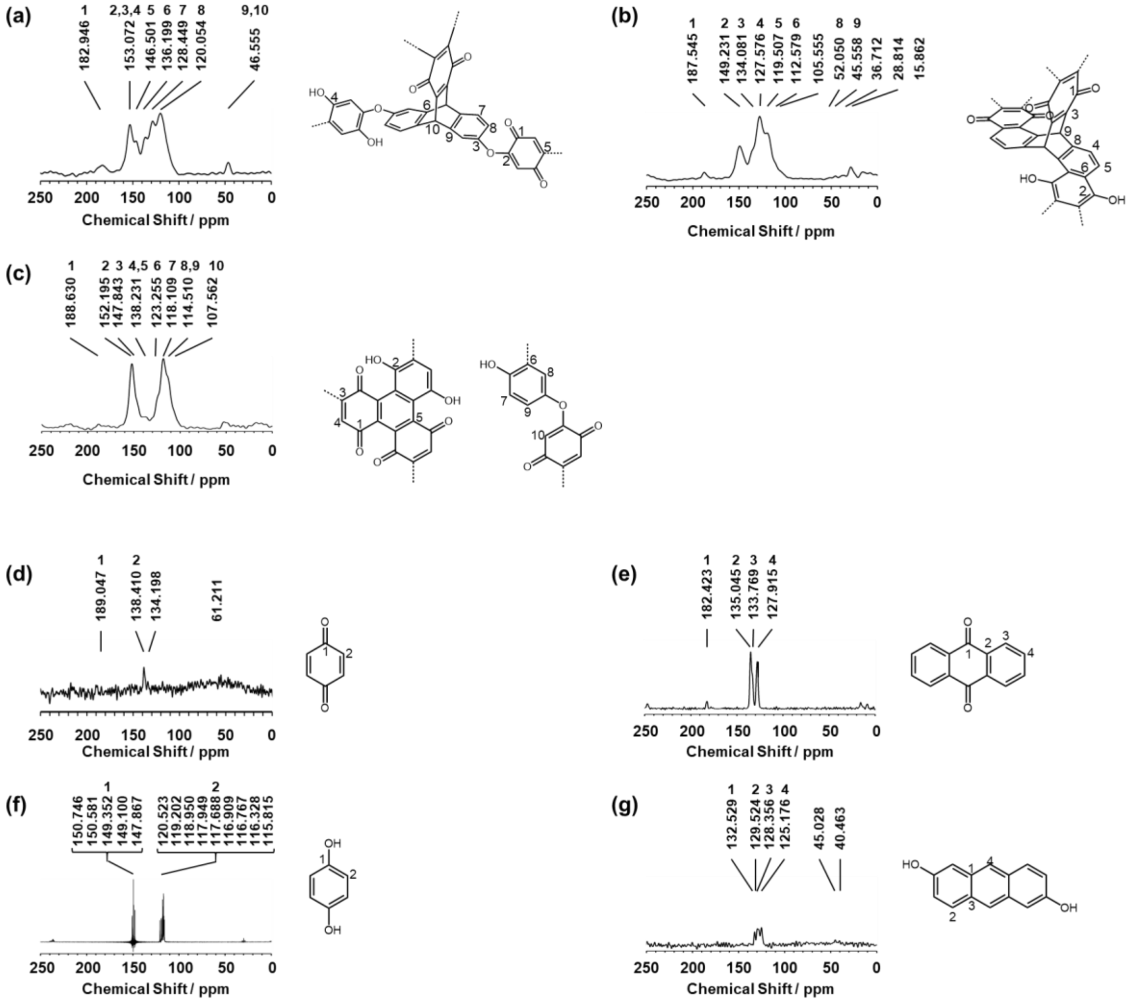

Fig. S3. ^{13}C solid-state NMR spectra of BQ-DAT (a), BQ-DVB (b), BQ-HQ (c), BQ (d), AQ (e), HQ (f), and DAT (g). ^{13}C solid-state NMR spectra were measured on the powdered samples by MAS.

The peaks are assigned to the carbons in the different states, as displayed with each spectrum. The peak broadening is ascribed to formation of the polymer. These results support formation of the BQ-DAT, BQ-DVB, and BQ-HQ polymers.

Estimated compositions

Table S2. Experimental and calculational compositions.

wt%	BQ-DAT			BQ-DVB			BQ-HQ		
	C	H	O	C	H	O	C	H	O
Experimental value	69.13	3.60	27.3	75.2	3.63	21.1	67.6	3.13	28.8
Calculated value	69.20	3.11	27.7	75.2	3.33	21.5	66.9	2.84	30.3

The experimental value was measured by CHN elemental analysis. The structure models with the composition in Fig. 3 was prepared on the basis of the experimental value. Then, the calculated value was estimated from the structure models to validate the experimental values.

UV-Vis-NIR spectra

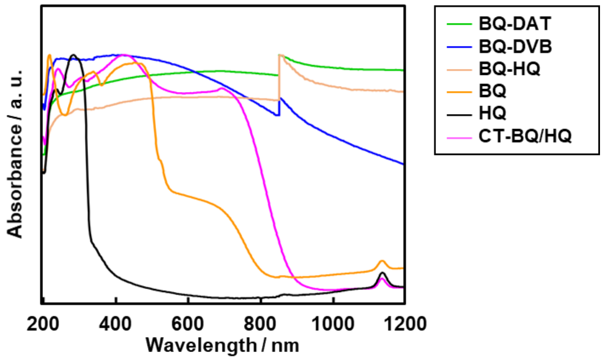

Fig. S4. UV-Vis spectra of BQ-DAT, BQ-DVB, BQ-HQ, BQ, HQ, and CT-BQ/HQ.

The BQ-DAT, BQ-DVB, and BQ-HQ polymers have broadened absorption in NIR region longer than 800 nm (green, blue, and yellow curves in Fig. S4). In contrast, the charge-transfer complex of BQ and HQ showed the absorption shorter than 900 nm (purple in Fig. S4). The results suggest that the absorption of the polymers in NIR region is ascribed to extension of the conjugation and formation of inter- and intramolecular CT complex between the BQ and HQ moieties. The step-like noises at 900 nm were caused by changes of the light source in spectrophotometer.

SEM images

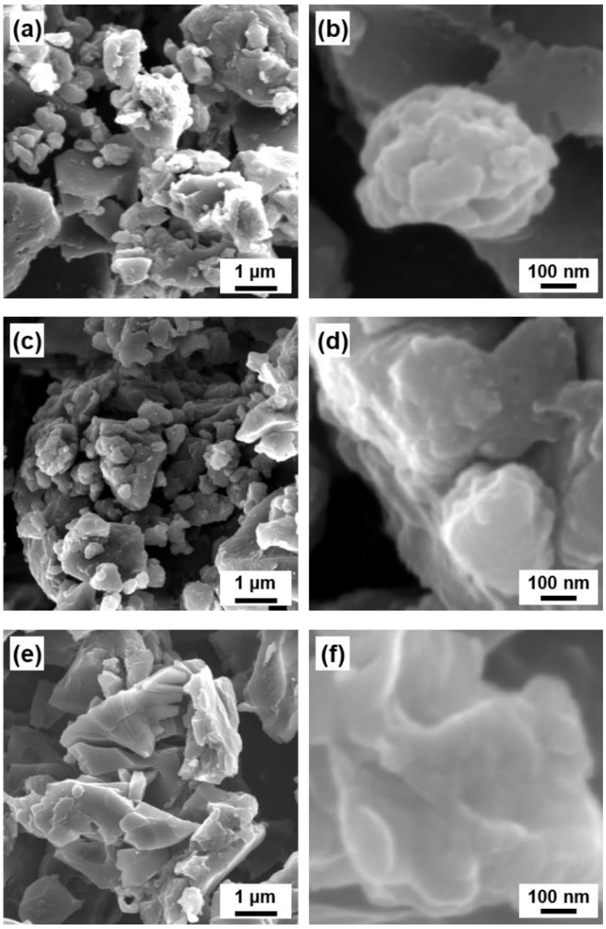

Fig. S5. SEM images of BQ-DAT (a,b), BQ-DVB (c,d), and BQ-HQ (e,f).

Irregular-shaped particles around 1 μm in size were observed on these polymers (Fig. S5a,c,e). The magnified images showed the primary particles, such as nanoflakes around 200 nm (Fig. S5b,d,f). These nanostructures are obtained by exfoliation treatment in organic dispersion media (Fig. 5).

AFM images, their height profiles, and thickness distribution

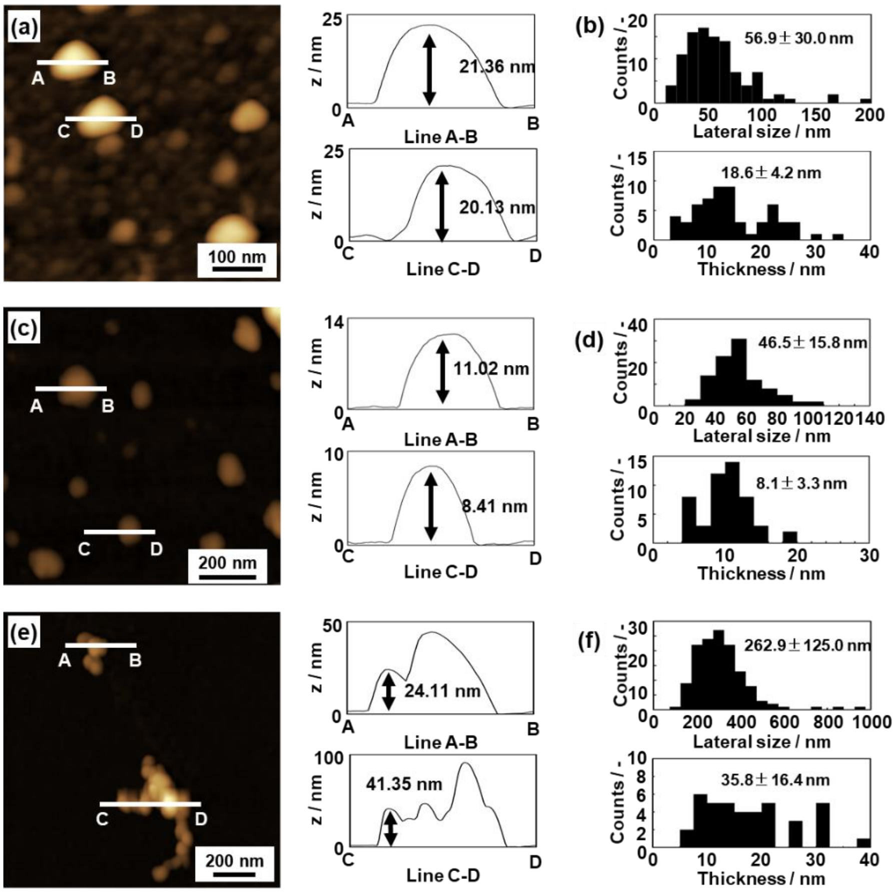

Fig. S6. AFM images, their height profiles, and thickness distribution of BQ-DAT (a,b), BQ-DVB (c,d), and BQ-HQ (e,f).

AFM images showed formation of the nanoflakes with the similar lateral size as observed in TEM and DLS charts (Fig. 5).

SAED patterns and HRTEM images

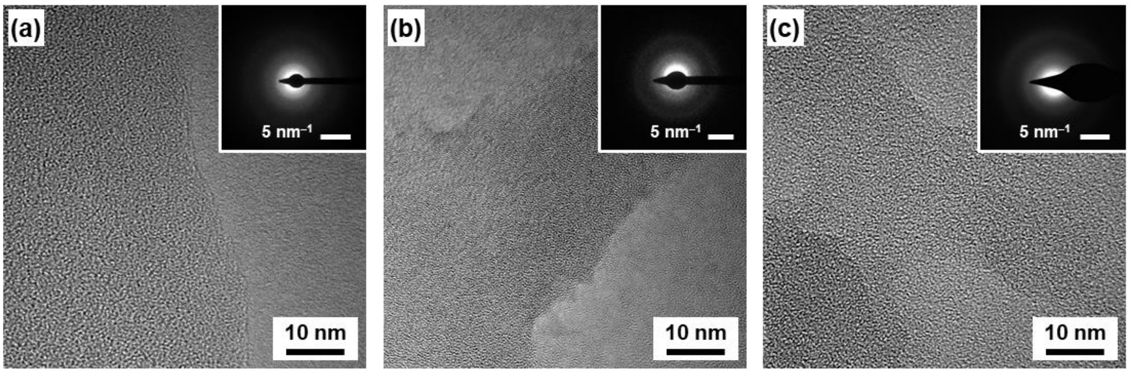

Fig. S7. HRTEM images and SAED patterns (the insets) of BQ-DAT (a), BQ-DVB (b), and BQ-HQ (c).

All the SAED patterns showed hallo without definite diffraction rings. No lattice fringes were observed on the HRTEM images. These results support the amorphous nature of the network polymers.

CV and charge-discharge curves

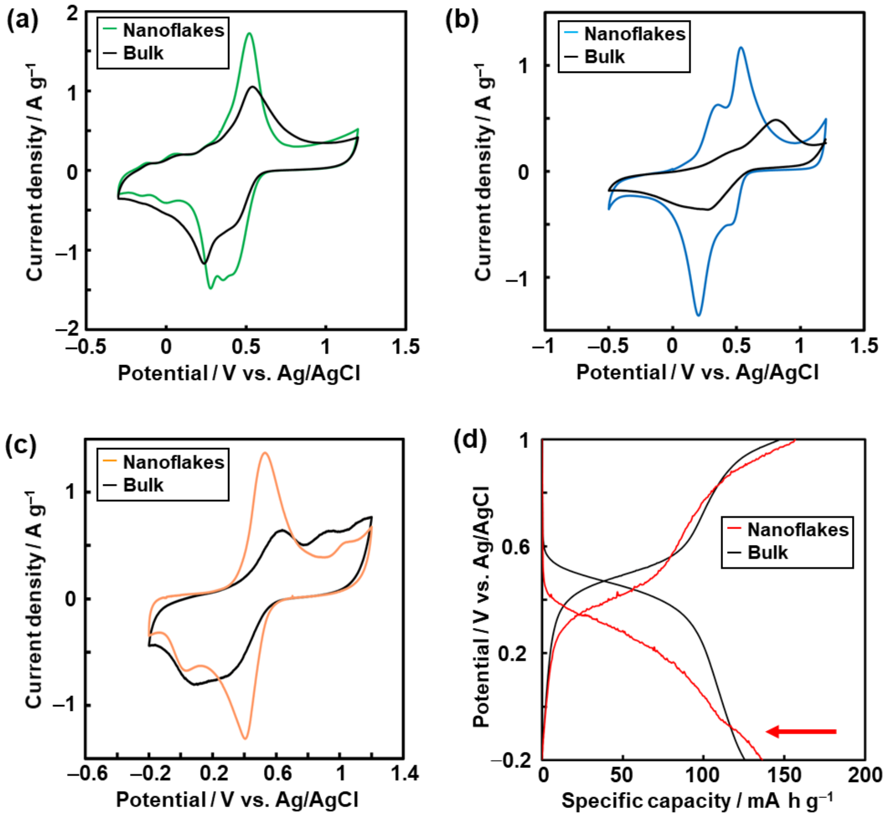

Fig. S8. CV (a–c) and charge-discharge (d) curves of the BQ-DAT (a), BQ-DVB (b), and BQ-HQ (c,d) polymers at 1 mV s^{-1} (a-c) and 0.2 A g^{-1} (d) before (the black curves) and after (the colored curves) the exfoliation treatment in dispersion media.

The current density was increased after the exfoliation treatment (Fig. S8a–c). The specific capacity was 155 mA h g^{-1} for BQ-DAT, 70.0 mA h g^{-1} for BQ-DVB, and 128 mA h g^{-1} for BQ-HQ on the bulky aggregates before the exfoliation. The specific capacity was improved to 176 mA h g^{-1} for BQ-DAT, 209 mA h g^{-1} for BQ-DVB, and 188 mA h g^{-1} for BQ-HQ after the exfoliation. The BQ-HQ nanoflakes showed the current originating from the side reactions at the potential around –0.2 V (the red arrow in Fig. S8d). In addition, the plateau was broadened on the nanoflakes because the BQ-HQ had the electrochemically unstable hexahydroxytriphenylene moiety in the network (Fig. 1i). Therefore, the charge-discharge curves of the BQ-HQ was obtained in the range –0.2–1.0 V on the sample before the exfoliation (Fig. 6c).

Reproducibility of the electrochemical properties

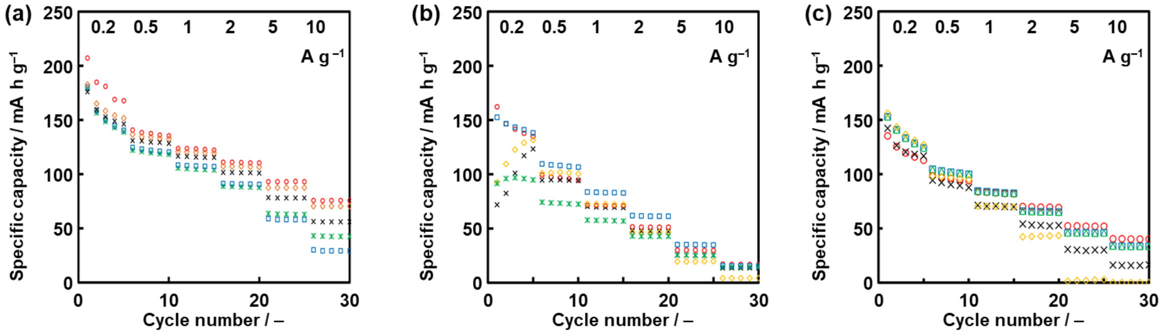

Fig. S9. Relationship between the current density and specific capacity of the BQ-DAT nanostructures (a), BQ-DVB nanostructures (b), and BQ-HQ (c) polymers before exfoliation with 5 different samples.

The measured capacity was reproducible. The slight differences in the mixed and coating states of the samples caused the data variation especially in the higher current density.

Effects of the branching on the electrochemical properties

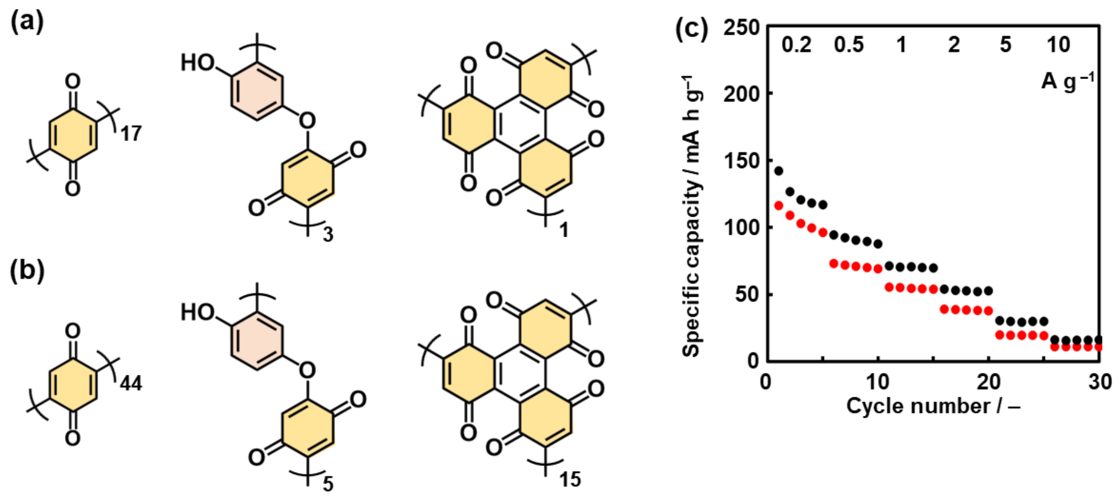

Fig. S10. Structures and electrochemical properties of the BQ-HQ polymers with the different branching states. (a,b) Estimated composition of the BQ-HQ polymers synthesized in H_2SO_4 at 25 °C (a) and by direct mixing of BQ and HQ at high temperature (b). Relationship between the current density and specific capacity of the BQ-HQ polymer (black) and the more branched one (red).

The reference BQ-HQ was synthesized by direct mixing of BQ and HQ in the absence of solvent at 190 °C for 48 h. The initial molar ratio was set to BQ:HQ = 7:3, whereas the BQ-HQ polymer was synthesized at the ratio BQ:HQ = 9:1 in H_2SO_4. The estimated composition based on CHN elemental analysis indicates that the more branched BQ-HQ polymer was obtained as the reference sample (Fig. S10b). The more branched BQ-HQ showed the lower specific capacity at all the current density (Fig. S10c). The results imply that the flexibility of the network structure plays an important role for penetration of the electrolyte.
